# Indoor air quality at the Arab governmental girls’ schools

**DOI:** 10.12688/f1000research.110775.1

**Published:** 2022-09-29

**Authors:** Mahmoud Fathy Elsharkawy, Mohammed Tawfiq Aljassim, Abdulmalik Salman Alsaif, Sana Abdullah Alsulaiman

**Affiliations:** 1 Department of Environmental Health, College of Public Health, Imam Abdulrahman Bin Faisal University, Dammam, Saudi Arabia; 2 Auxiliary Agency for Preventive Health, Ministry of Health of Saudi Arabia, Riyadh, Saudi Arabia

**Keywords:** School environment, Indoor air quality, Air quality guidelines, Girl schools, Saudi Arabia

## Abstract

**Background:** A proper and adequate school environment is important for an effective learning process and maintaining the health of the students as they spend most of their time in schools. The physical school environment includes the physical structures; presence of chemicals and biological agents; and the surrounding environment, including air, water, and materials.

This study aimed to evaluate the indoor air quality (IAQ) in governmental girls’ schools in the Kingdom of Saudi Arabia (KSA).

**Methods**: Seventeen girls’ schools were randomly selected in the Eastern Province of KSA. The indoor levels of PM _10_ and PM _2.5_, volatile organic compounds, CO, NO _2_, and CO _2_ were measured at different sites inside each school during four months of the year 2020. In addition, a pre-designed questionnaire was used to evaluate the safety preparedness in the same selected schools.

**Results:** Levels of the six air pollutants were higher inside schools adjacent to roads with moderate traffic activity than schools with low and very low traffic activity. However, the mean level of CO _2_ inside the selected schools was higher than its outdoor level, suggesting the predominance of an indoor source of CO _2_. Levels of all measured air pollutants inside governmental constructed school buildings were higher than those inside the rental type.

**Conclusion:** The average levels of air pollutants inside the selected schools were much lower than their air quality guidelines (AQGs), while some CO _2_ and NO _2_ levels exceeded their AQGs at some schools.

## Introduction

A proper and adequate school environment is important for an effective learning process and maintaining the health of the students because they spend most of their daytime in schools. ^1^^–^^3^ The effectiveness of the school depends on the quality of its environment and its ability to encourage students to learn effectively. ^4^ The physical school environment includes the type of buildings, internal infrastructure, furniture, presence of chemicals or biological agents, location, and its surrounding environment, which includes air, water, and nearby land uses, roadways, and other sources of contaminants. ^5^^,^^6^

Inside schools, there are many sources of air pollution such as the building materials, furnishing, wall paints, air conditioning system, cleaning products, and chemicals that are used in laboratory, as well as the transformation of pollutants from the outdoor sources and surrounding activities (e.g., traffic roads, industry, agricultural or commercial). ^7^

Recently, the “green school concept” has been strongly recommended for schools, particularly new ones. A green school is defined as “a school that creates a healthy environment conducive to learning through building the school with more daylighting, better ventilation, healthy green building materials (such as non-VOC (volatile organic compound) carpets and paints), and planting more trees and plant around and inside the school premises to reduce carbon emissions and other air pollutants”. ^8^ Respiratory conditions, such as asthma, are a major cause of hospitalization and school absenteeism in several countries across the world. ^9^^,^^10^ In addition, students and school personnel might experience emergency medical situations because of injuries or unexpected major accidents that occur in schools due to the absence of the recommended control measures or safety preparedness. ^11^

Poor indoor air quality (IAQ) may increase rates of asthma, allergies, and infectious respiratory diseases, affecting student performance. ^12^^,^^13^ Poor IAQ, including inadequate ventilation, air pollutants, and very high or very low temperatures, is one of the factors that can contribute to absenteeism and reduce the performance of both students and staff if not adequately considered and controlled. ^14^ Furthermore, schools in industrialized countries or high traffic areas are subjected to higher indoor air pollution than those in rural or low traffic ones. The good IAQ test is one of the key factors contributing to a healthy and productive learning environment. ^15^^–^^19^

Although several studies have been conducted to evaluate the IAQ and safety preparedness of the boys’ schools in KSA, no previous similar studies have been conducted for the girls’ schools. The major importance of this study is the formation of a database for environmental quality, particularly air quality, and safety preparedness inside the girls’ schools in KSA. In addition, this study will help in raising awareness of students and all school personnel concerning the safety and environmental health for prevention or reducing the exposure to the risks, accidents, and environmental disasters that have existed in the schools.

## Methods

### Ethical considerations

This research was approved by the Institutional Review Board (IRB) of Imam Abdurrahman bin Faisal University No. IRB-PGS-2019-03-357 at 4/12/2019. Because our study was on the governmental schools, we obtained the necessary approvals from the Saudi Ministry of Education prior to the completion of this study. A general consent from the General Educational Administration in the Eastern Province of the Kingdom was obtained, which gave us the freedom to enter any school without the need to obtain its separate consent. Our role was to coordinate with the selected school before going to conduct the measurements and collect the required data, which was usually done by a phone call or a quick visit.

### Study site and duration

A cross-sectional descriptive study was conducted for measuring the levels of air pollutants in 17 selected schools at Al-Qatif governorate during a period of four months (January – April 2020). Al-Qatif is an urban area with an ancient green coastal oasis rich in oil, dates, fruits, and fish, surrounded by a jungle of palm trees. The climate of Al-Qatif is hot and dry with high humidity and temperatures in summer and cold in winter. Its population is 524,182 people, according to the statistics of 2017. ^20^ The traffic activity in Al-Qatif is not congested compared to other cities of the Eastern Province of KSA.

### Details of the studied schools

Generally, a good maximum sample size is usually around 10% of the total population. To increase the credibility of our work, we selected seventeen girls’ schools were selected. This number represents nearly 20% of the KSA governmental girls’ schools in Al-Qatif governorate. Selection of this number of schools was based on a completely randomized design (CRD), which is considered one where the treatments are assigned completely at random so that each experimental unit has the same chance of receiving any one treatment. The process of random allocation may be done in several ways. To select our studied schools, we were using drawing lots through giving numbers for all schools (80 schools) and then drawing 17 number randomly. The inclusion criteria were the KSA governmental girls’ schools in Al-Qatif governorate, while the exclusion ones were the governmental boys’ schools and private boys’ and girls’ schools in the same governorate. Details of the selected schools, including the type of school building, outside traffic activity, number of classrooms and floors, and the total number of students and staff, are presented in Table 1.

**Table 1.** Detail of the selected schools for this study.

No. of school	Type of school building	Outside traffic activity	Total No. of classrooms	Total No. of students	Total No. of staff	Number of floors
1	Governmental	Moderate	27	492	48	2
2	Governmental	Moderate	29	379	35	2
3	Governmental	Low	55	509	57	3
4	Governmental (Aramco)	Moderate	50	763	71	1
5	Governmental	Very low	16	149	27	2
6	Governmental	Very low	55	1035	87	3
7	Governmental	Low	32	383	40	3
8	Governmental	Very low	24	273	39	2
9	Governmental	Very low	24	367	58	2
10	Government	Very low	22	283	37	3
11	Government	Very low	22	295	55	2
12	Governmental	Moderate	32	150	33	2
13	Governmental (Aramco)	Moderate	41	481	51	2
14	Governmental	Moderate	43	265	58	3
15	Rented	Low	17	312	37	3
16	Rented	Very low	40	518	46	4
17	Rented	Very low traffic	24	414	37	3

To study the effect of traffic emissions on the IAQ level outside schools in this study, the traffic activity was classified into three categories based on the average number of cars that were moving per hour. ^21^ The first category is “very low traffic activity” where the number of cars was <50 cars per hour. The second category is “low traffic activity” where the number of cars ranged from 50 to <200 cars per hour. The third category is “moderate traffic activity” where the number of cars ranged from 200 to <500 cars per hour.

### Monitoring of IAQ

Inside each school, several locations were selected for monitoring of the air quality measurements. These locations represented two classrooms from each floor, the playground, the library, and the laboratory. Inside each one of these rooms, two sites were selected; at the front and back of the room. The total number of selected locations ranged between 14 and 22 based on the number of floors inside each school. Levels of particulate matter less than 2.5 and 10 microns in size (PM _10_ and PM _2.5_, respectively), volatile organic compounds (VOCs), carbon monoxide (CO), carbon dioxide (CO _2_), and nitrogen dioxide (NO _2_) were monitored inside each one of the selected locations at each school. In addition, temperature and relative humidity (RH) percent were simultaneously recorded for all locations.

Levels of PM _10_ and PM _2.5_ were determined by the Handheld Portable Particle Counter instrument (M&A Instruments Inc). It can measure directly PM _10_ and PM _2.5_ in a very wide range of concentrations (0-500 μg/m ^3^) with a high degree of accuracy, because it automatically monitors the sensor status, out of flow calibration (> 5%). In addition, it has external digital temperature and humidity sensors to assure accurate measurement.

Generally, levels of gaseous air pollutants are measured by direct-reading instruments based on different techniques such as infrared, ultraviolet, or electrochemical techniques. During this study, all selected gases were directly measured by the Gray Wolf’s Direct Sense ^®^ mobile PC based products, Advanced Sense™ meters, and Wolf Pack™ area monitor. The Gray Wolf monitor is composed of multi-gas detectors. It is a one to five-sensor gas detector equipped with a wireless radio frequency modem which allows the unit to communicate and transmit readings and other information on a real-time basis with a remotely located base controller. In stand-alone operation, it is a rugged, weather-resistant, portable monitor that can run over 24 hours on either rechargeable lithium-ion or alkaline batteries. In addition, it has sensors from measuring the ambient temperature degree and RH percent.

At each measuring site inside the school, a reading for each selected pollutant was recorded every 30 minutes during a total of 2 hours period. The gaseous pollutants were measured directly in parts per million (ppm), while levels of PM _10_ and PM _2.5_ were recorded directly in microgram of dust per cubic meter of air (μg/m ^3^). Levels of the selected air pollutants were measured at least three times during the morning period of different weekdays where students and staff are present in their classrooms. The total number of IAQ readings nearly ranged between 30 to 50 for each pollutant for each school.

### Statistical analysis

Results of IAQ monitoring were analyzed statistically using professional programs such as the Statistical Package for the Social Sciences (SPSS) version 23 and Excel Software 2016. Descriptive statistics, ANOVA test and Pearson correlation coefficient were used for comparing levels of the studied air pollutants at different sites inside and outside the selected schools. The statistical significance of p-value <0.05 was used for all tests of significance.

## Results

Although about five measurements were conducted for each type of pollutant at each selected site inside the schools, we only considered the most two stable measurements in our analysis. ^63^ All selected schools were eligible for the study and no problems or constrains were faced during the study. Table 2 represents the mean levels and standard deviations (SD) of PM _10_, PM _2.5_, CO, CO _2_, VOCs, and NO _2_, respectively, at all selected schools. The highest mean levels ± standard deviation (S.D) for PM _10_ and PM _2.5_ were (29.3 ± 6.5 μg/m) and (16.3 ± 1.3 μg/m), where the lowest mean levels were (12.0 ± 2.2 μg/m) and (7.5 ± 1.3 μg/m) respectively. The highest mean levels of CO _2_, CO, NO _2_, and VOCs were (1488 ± 533.3 ppm), (3.85 ± 0.66 ppm), (1.32 ± 0.2 ppm) and (0.34 ± 0.47 ppm) respectively, while the lowest mean levels were (523.0 ± 85.3 ppm), (1.2 ± 0.1 ppm), (0 ppm) and (0 ppm) respectively.

**Table 2.** Mean levels of air pollutants inside the studied schools (VOCs: volatile organic compounds).

No. of school	Results	PM _10_ (μg/m ^3^)	PM2.5 (μg/m ^3^)	CO _2_ (ppm)	CO (ppm)	NO _2_ (ppm)	VOCs (ppm)
1	Mean	29.3	15.8	1107.4	3.85	1.32	0.3
	SD	6.5	2.3	539.2	0.66	0.20	0.04
2	Mean	17	11.1	723.5	3.73	0.58	0.23
	SD	7.1	3.6	147	0.77	0.15	0.03
3	Mean	17.3	8.8	531.8	3.7	0.28	0.16
	SD	4.3	1.6	92.7	2.61	0.02	0.03
4	Mean	21.9	14.4	1488	2.86	0.73	0.29
	SD	5.4	1.6	533.3	0.52	0.50	0.04
5	Mean	16.3	11.6	691.3	2.95	0.56	0.10
	SD	4.1	2.2	119.3	2.29	0.78	0.06
6	Mean	15.2	8.5	702.5	1.93	0	0.34
	SD	0.5	4.1	162.4	0.12	0	0.47
7	Mean	15	12.8	580.3	2.73	0	0
	SD	5.1	3.1	143.2	0.81	0	0
8	Mean	12	9	614.8	2.63	0	0
	SD	2.2	1.8	171.8	0.87	0	0
9	Mean	14.3	12	523	2.2	0	0
	SD	1.3	1.83	85.3	0.36	0	0
10	Mean	18	9.5	672.8	2.9	0	0
	SD	3.6	2.1	254.4	0.69	0	0
11	Mean	16.8	12.3	601.8	2.23	0	0
	SD	2.4	2.2	119.4	0.46	0	0
12	Mean	28.8	14	951.8	2.05	0	0.25
	SD	2.1	0.8	425.9	0.31	0	0.03
13	Mean	23.8	13.8	685	1.58	0.91	0
	SD	0.9	2.2	197.4	0.33	0.74	0
14	Mean	24.8	16.3	1019.8	1.86	0.915	0.28
	SD	4.5	1.3	451.6	0.28	0.41	0.04
15	Mean	20.8	13.5	669.5	1.2	0.23	0
	SD	2.2	1.7	219.2	0.1	0.32	0
16	Mean	14.8	7.5	653.3	2.6	0.25	0
	SD	2.8	1.3	245.7	1.29	0.08	0
17	Mean	15.5	11.5	561	2.13	0.38	0
	SD	3.8	1.3	103.6	0.93	0.17	0

**Figure 1.**
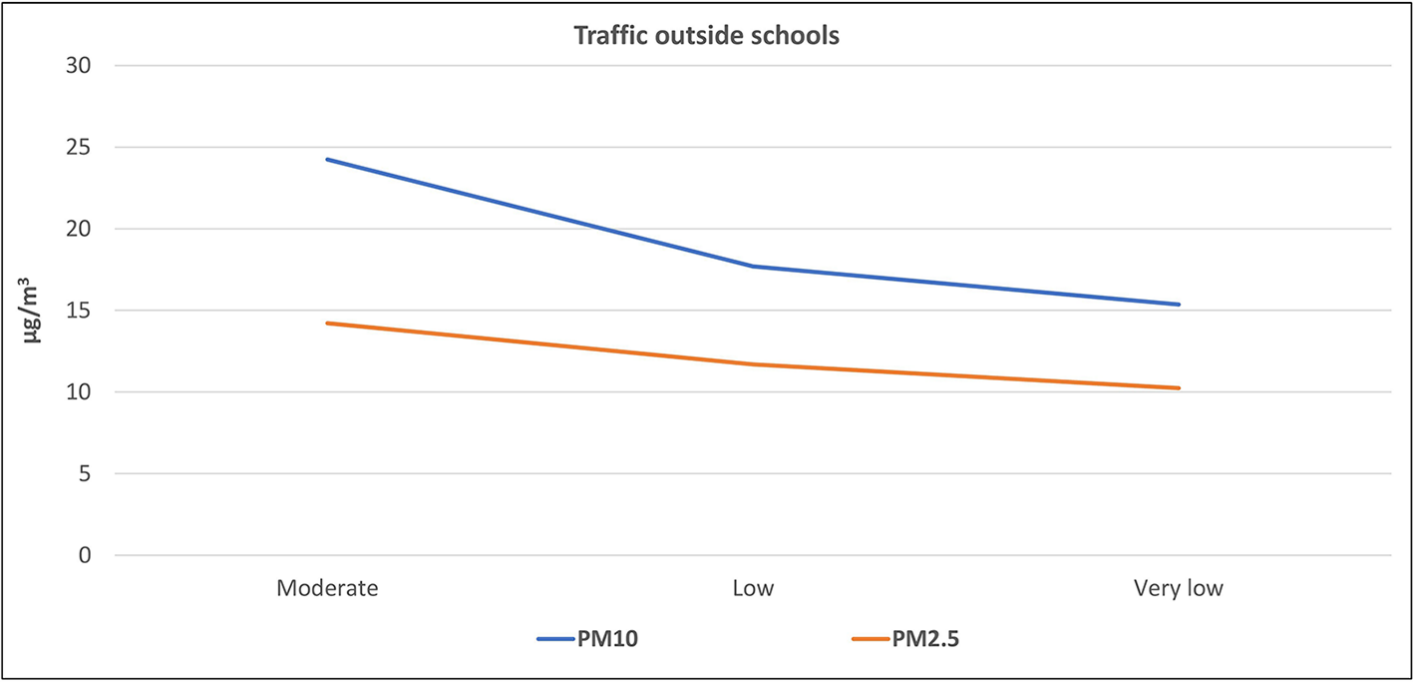
Relation between levels of PM inside schools and the outdoor traffic activity.

**Figure 2.**
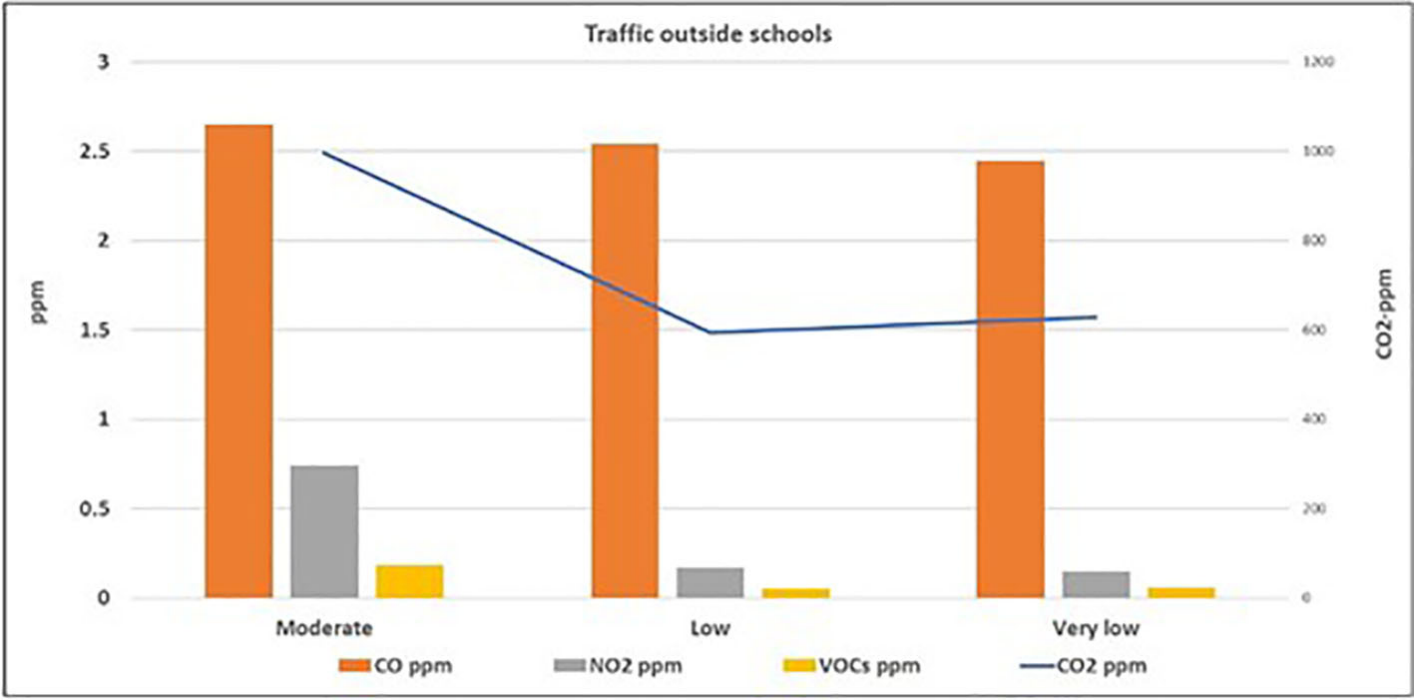
Relation between levels of gaseous air pollutants inside schools and the outdoor traffic activity.

Figures 1 and 2 illustrate the correlation between the level of air pollutants inside the selected schools and the traffic activity outside the schools. The highest levels (mean ± standard deviation) of PM _10_ (24.2 ± 4.6 μg/m ^3^), PM _2.5_ (14.2 ± 1.8 μg/m ^3^), CO (2.65 ± 0.99 ppm), CO _2_ (995.9 ± 292.6 ppm), VOCs (0.18 ± 0.14 ppm), and NO _2_ (0.74 ± 0.44 ppm) were found inside schools surrounded by streets with moderate activity, followed by those located adjacent to the streets with low traffic activity, while the lowest levels were found inside the schools surrounded by streets with very low activity. On the contrary, the mean level of CO _2_ in schools adjacent to the very low traffic street (627.5 ± 63.9 ppm) was slightly higher than those at the low traffic street (593.9 ± 69.8 ppm). To study the significance of this factor, the one-way ANOVA test was applied for the data, as presented in Table 3 which indicates that significant differences (p < 0.05) were found between schools located at moderate streets and those in low and very low ones for four pollutants, except for CO and VOCs.

**Table 3.** One-way ANOVA test between air pollutant levels inside schools and outdoor traffic activity.

Dependent variable	Location of schools		Std. error	Sig. (P value)
PM _10_ (μg/m ^3^)	Very low traffic	Low traffic	1.774	.241
		Moderate traffic	1.212	.000 *
	Low traffic	Moderate traffic	1.728	.001 *
PM _2.5_ (μg/m ^3^)	Very low traffic	Low traffic	.878	.373
		Moderate traffic	.600	.000 *
	Low traffic	Moderate traffic	.855	.002 *
CO _2_ (ppm)	Very low traffic	Low traffic	114.9988	.632
		Moderate traffic	78.5592	.000 *
	Low traffic	Moderate traffic	111.9777	.000 *
CO (ppm)	Very low traffic	Low traffic	.41239	.696
		Moderate traffic	.28278	.054
	Low traffic	Moderate traffic	.40023	.332
NO _2_ (ppm)	Very low traffic	Low traffic	.14619	.746
		Moderate traffic	.09987	.000 *
	Low traffic	Moderate traffic	.14235	.000 *
VOCs (ppm)	Very low traffic	Low traffic	.69772	.995
		Moderate traffic	.47663	.196
	Low traffic	Moderate traffic	.67939	.366

* The mean difference is significant at the 0.05 level.

**Figure 3.**
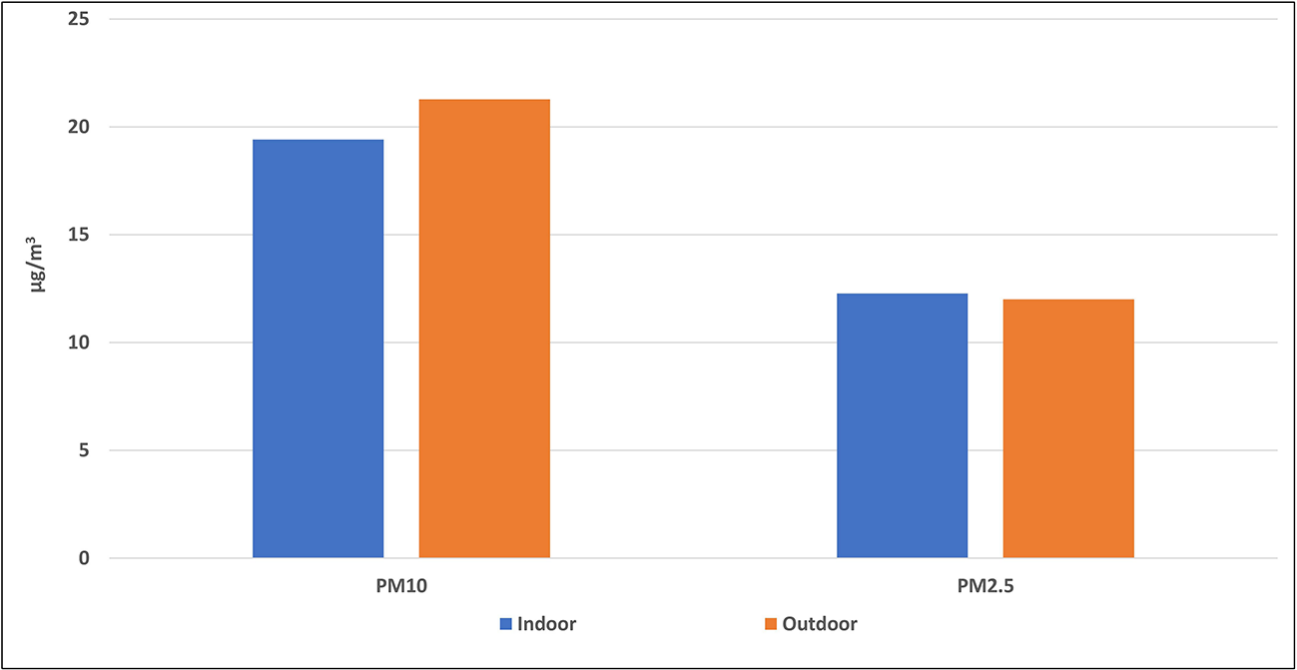
Relation between indoor and outdoor levels of PM.

**Figure 4.**
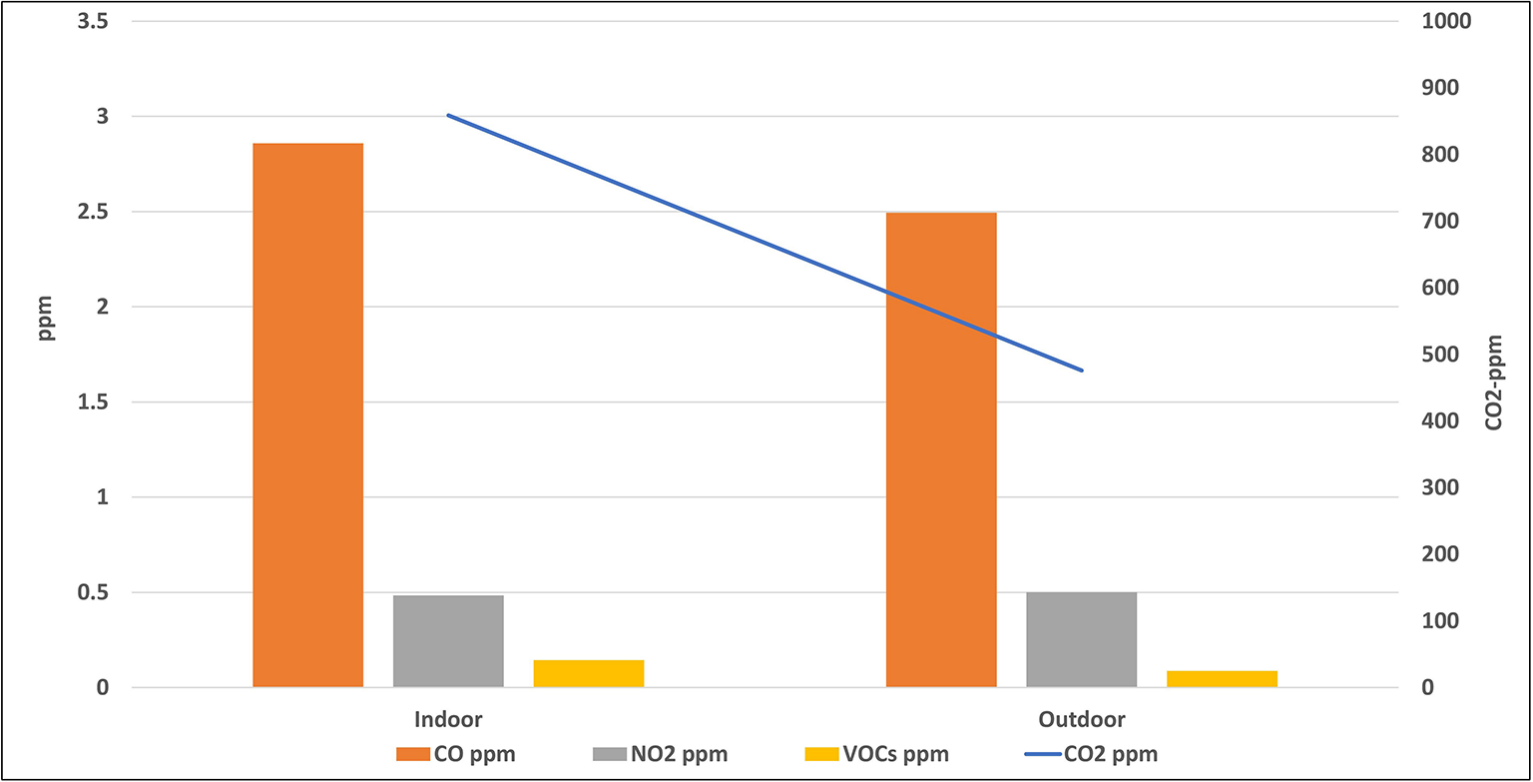
Relation between indoor and outdoor levels of gaseous air pollutants.

Figures 3 and 4 indicate the average concentrations of outdoor air pollutants compared with those inside schools. Except for CO _2_, the average concentrations of all pollutants outside schools were slightly higher or nearly the same as the indoor levels, with no statistical differences for all pollutants (p > 0.05). On contrary, the mean level of CO _2_ inside the selected schools (858.7 ± 436.7 ppm) was higher than its outdoor level (475.9 ± 116.8 ppm) with a very strong statistical difference (p = 0).

**Figure 5.**
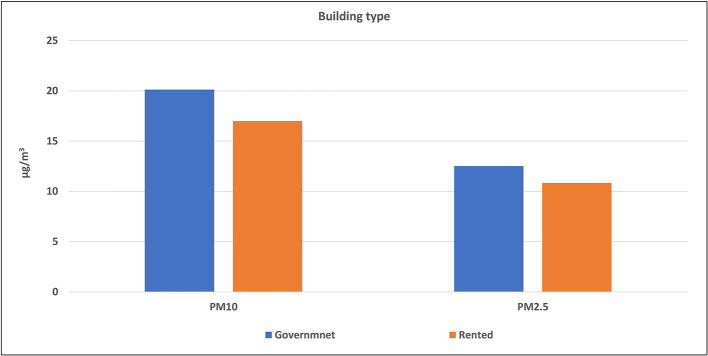
Levels of PM inside schools of different building types.

**Figure 6.**
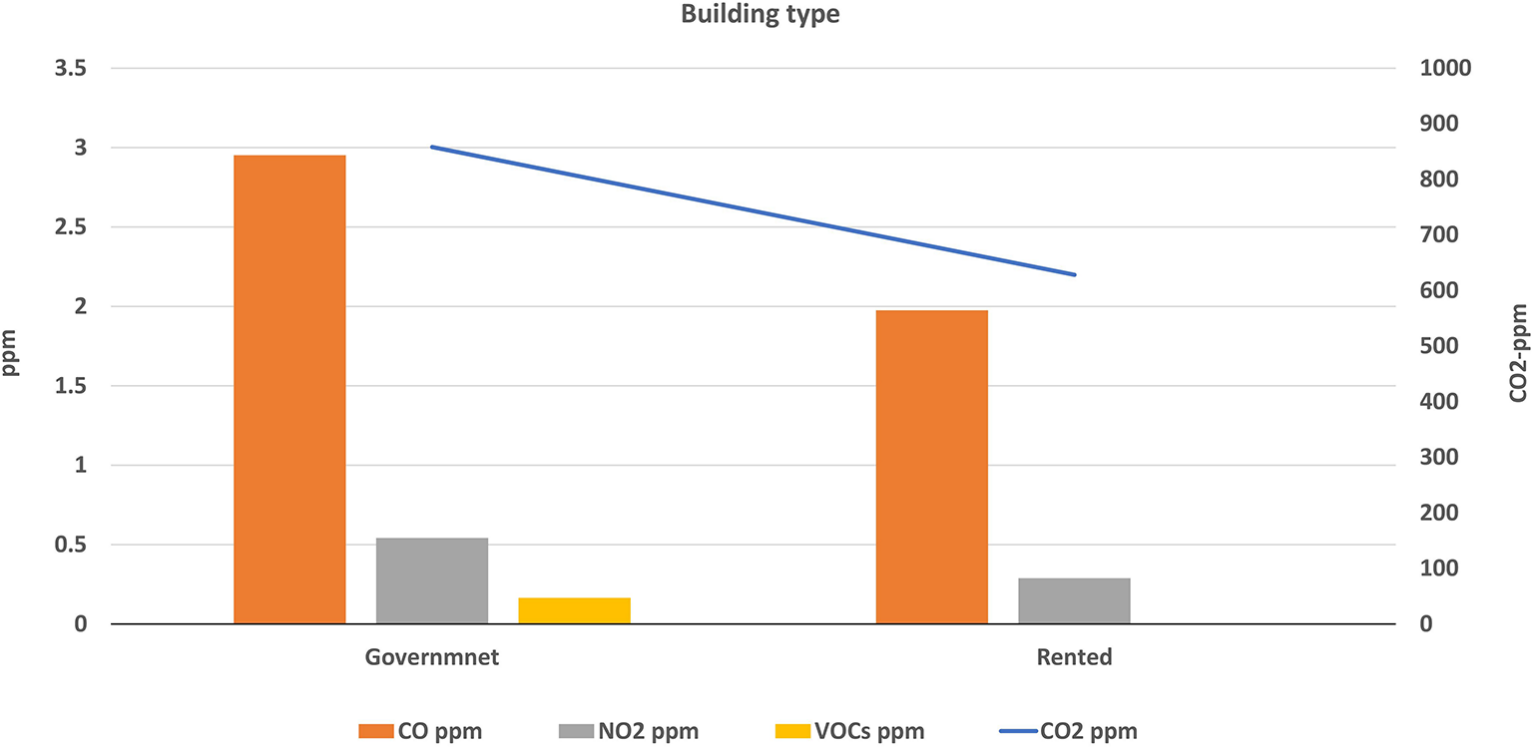
Levels of gaseous air pollutants inside schools of different building types.

Buildings of the selected schools were divided into two types: governmental constructed buildings (which were 14 schools) and governmental rental buildings (3 schools). The average levels of air pollutants inside each type were calculated and illustrated in Figures 5 and 6. The mean concentrations of studied pollutants inside the governmental constructed schools’ buildings (PM _10_ [20.1 ± 7.1 μg/m ^3^], PM _2.5_ [12.5 ± 3.3 μg/m ^3^], CO [2.95 ± 1.33 ppm], NO _2_ [0.54 ± 0.58 ppm], CO _2_ [858 ± 440.8 ppm] and VOCs [0.16 ± 0.15 ppm]), were higher than those inside the rental type (17 ± 3.9 μg/m ^3^, 10.8 ± 2.9 μg/m ^3^, 1.98 ± 1.03, 0.29 ± 0.20, 628 ± 187 and 0 ± 0 ppm respectively). The independent t-test values ( Table 4) indicated a statistically significant difference for CO, CO _2_, and NO _2_ levels (p < 0.05) between governmental constructed and rental school buildings, while there is no significance for the other pollutants. The independent t-test values ( Table 4) indicated a statistically significant difference for CO, CO _2_, and NO _2_ levels (p < 0.05) between governmental constructed and rental school buildings, while there is no significance for the other pollutants.

**Table 4.** T-test between air pollutants levels in the governmental constructed and rental school buildings.

Independent samples test	t-test for equality of means		
	t	df	Sig. (2-tailed)
PM _10_	2.322	22.155	.030
PM2.5	1.845	14.892	.085
CO _2_	3.245	30.756	.003 *
CO	2.976	16.359	.009 *
NO _2_	2.986	41.176	.005 *
VOCs	1.655	91.000	.101

* The mean difference is significant at the 0.05 level.

**Figure 7.**
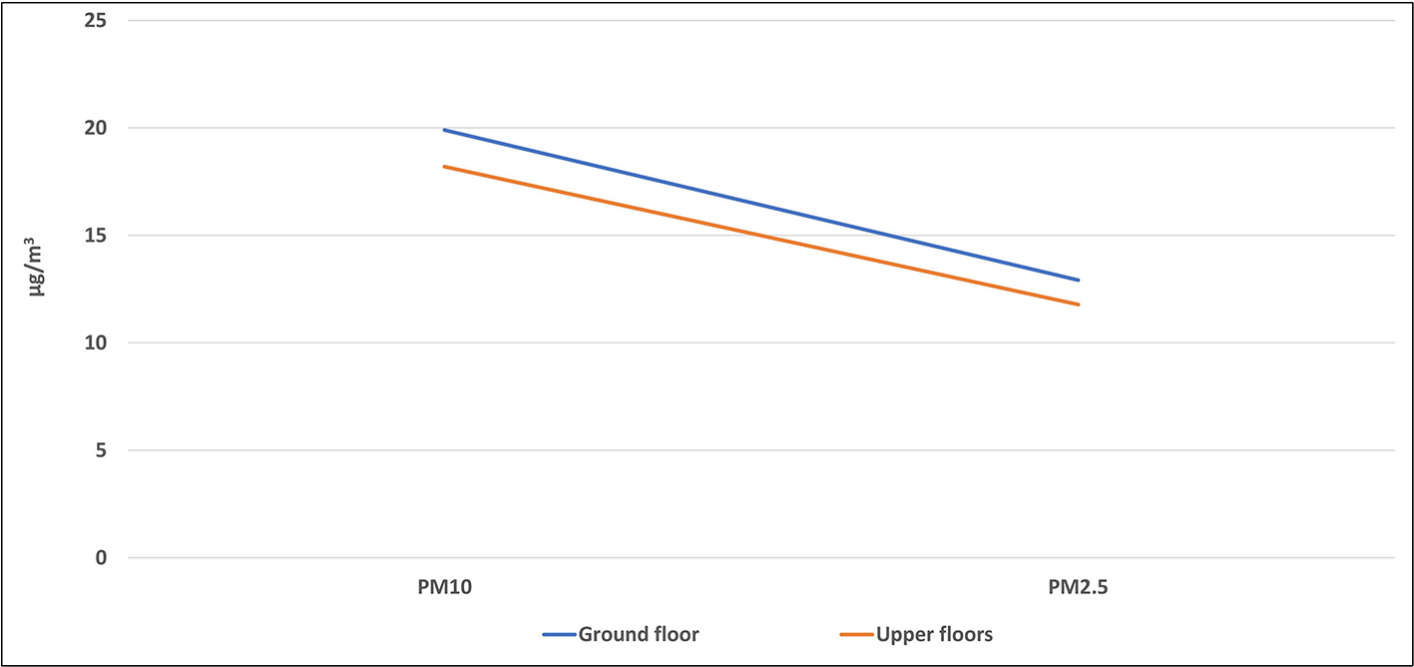
Levels of PM inside classrooms located on different floors.

**Figure 8.**
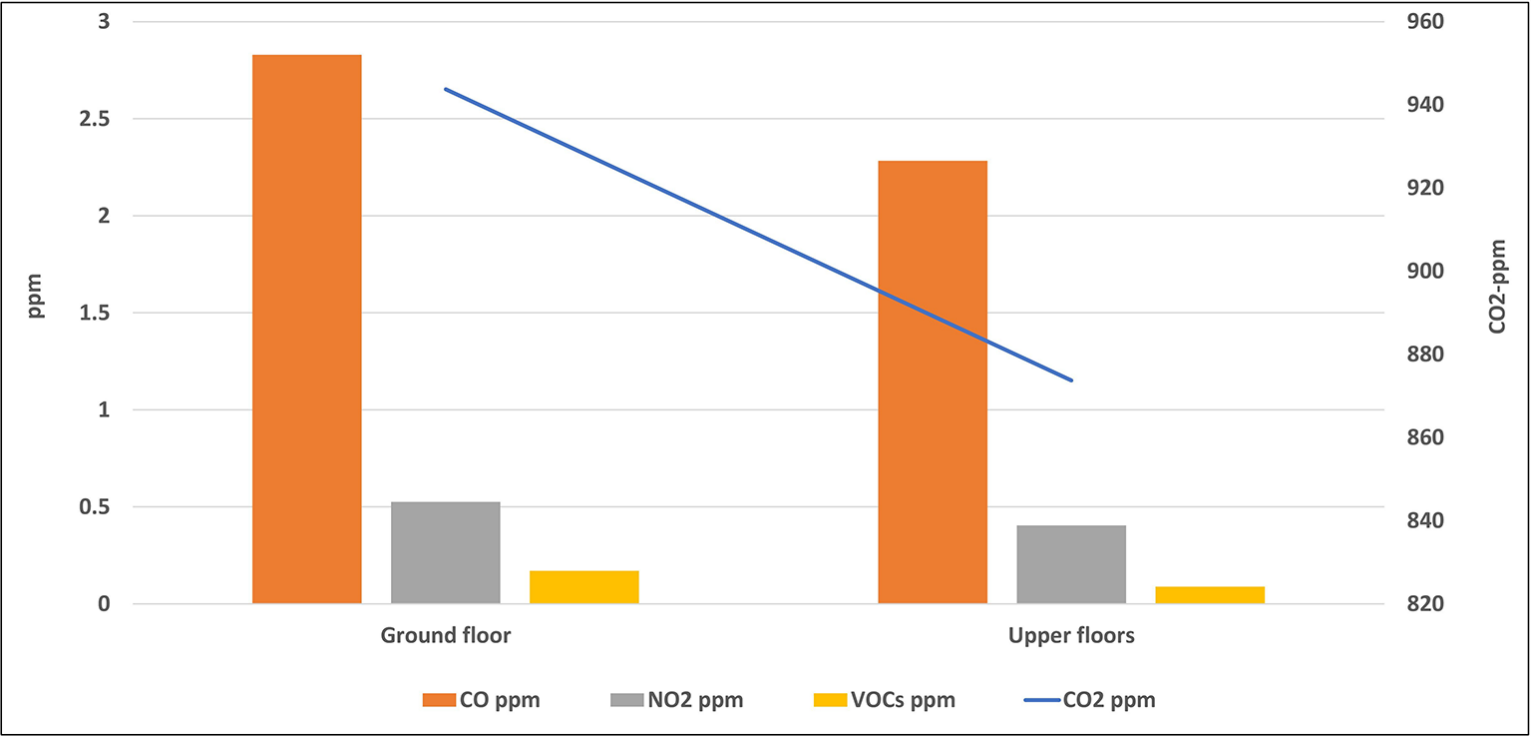
Levels of gaseous air pollutants inside classrooms located on different floors.

Some of the studied classrooms for this study were located at the ground level (ground floor), while the others were located on the upper floors. The average level of each air pollutant inside all classrooms of the same floor was calculated and presented in Figures 7 and 8.

High levels of CO _2_ (˃ 1000 ppm) were found in schools of both large and small areas, while low levels (< 500 ppm) were also recorded in schools with both large and small areas. Using the Pearson correlation tests indicated that there is a negative very weak correlation between the total volume of classrooms and levels of CO _2_ with no significant correlation (P > 0.05).

**Figure 9.**
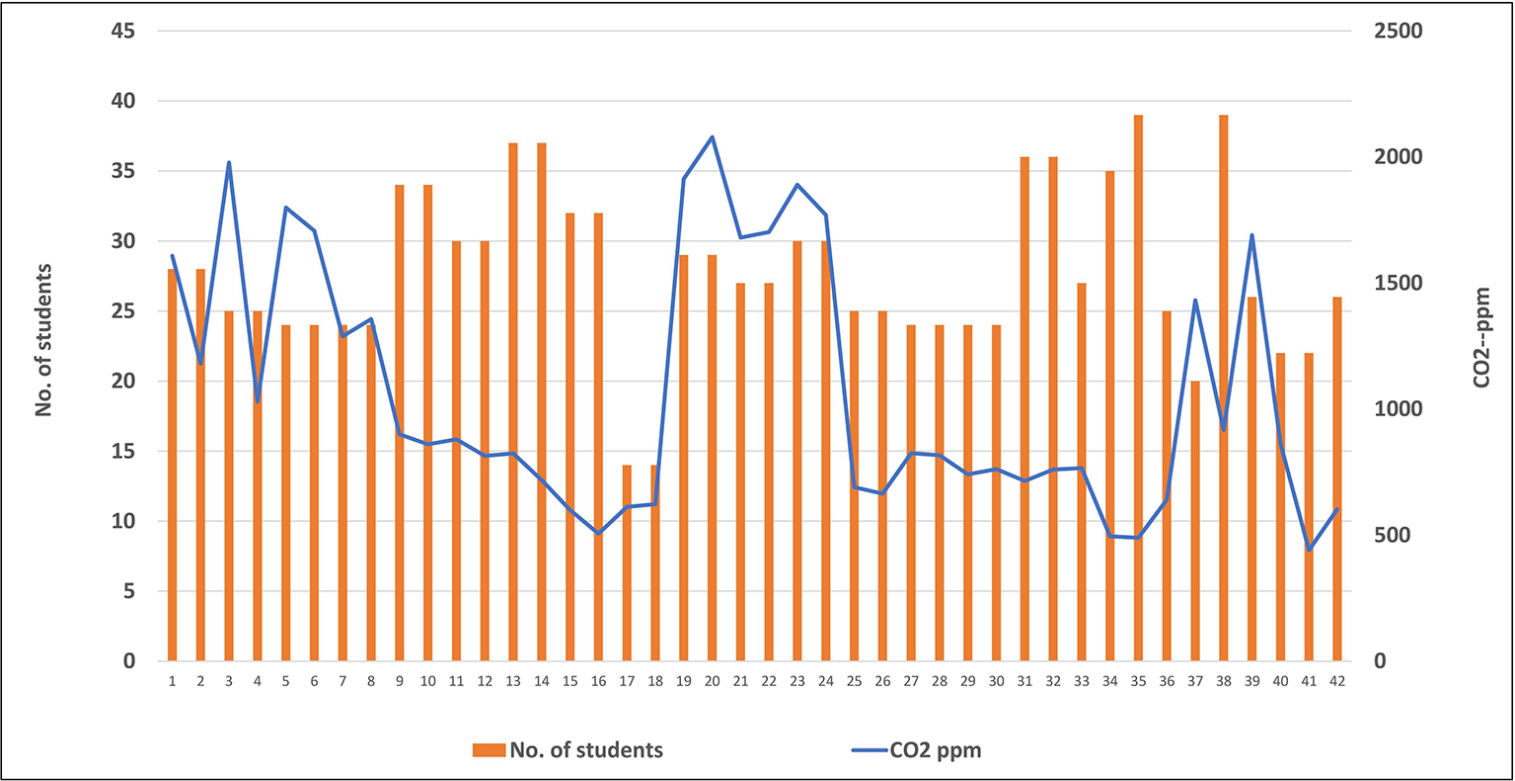
Relation between levels of CO _2_ and the number of students in classrooms.

Figure 9 represents the relation between levels of CO _2_ and the number of students in classrooms, where no correlation was found between these two variables. For example, lower level of CO _2_ (703.5 ppm) was found inside classrooms occupied with high number of students (39 students), while higher level of CO _2_ (1382.7 ppm) was obtained inside classrooms occupied with lower number (27 students).

The Pearson correlation coefficient was used to study the correlation between concentrations of the studied pollutants and degree of temperature in all selected classrooms, as shown in Table 5. There is a very weak positive correlation between the degree of temperatures and concentrations of both PM _10_ and VOCs, while there is a very weak negative correlation with levels of the other pollutants without statistical significance for all pollutants (p ˃ 0.05).

**Table 5.** Pearson correlation test between levels air pollutants and the temperature degree and RH percent inside classrooms.

Item	Type of correlation	PM _10_	PM _2.5_	CO _2_	CO	NO _2_	VOCs
Temperature degree	Pearson correlation	.174	-.169	-.068	-.125	-.075	.114
	Sig. (2-tailed)	.077	.085	.495	.209	.446	.250
RH percent	Pearson correlation	.328	.434	.491	.263	.388	-.127
	Sig. (2-tailed)	.001 *	.000 *	.000 *	.007 *	.000 *	.197

* The mean difference is significant at the 0.05 level (RH: relative humidity).

## Discussion

Occupants of an indoor environment, including homes, workplaces, and schools are exposed to a mixture of pollutants with known health effects, such as VOCs, CO, PM _10_ and PM _2.5_, and other gases. The contribution and concentration of these pollutants are influenced by various sources, for example, indoor sources, outdoor air pollution and climate, occupant’s behavior, and building material. ^22^^,^^23^ In the present study, most of these factors were studied in the selected schools to determine the factor that mostly affects school and classroom IAQ levels.

### Effect of the outdoor traffic activity on the IAQ levels

Motor vehicles emit many pollutants that can cause adverse health effects. ^24^ Children are exposed to pollutants in school- related spaces for many hours of the day. In general, schools in downtown areas are exposed to pollutants from traffic emissions higher than those located in low traffic areas. ^25^ Evidently, the levels of the six studied air pollutants inside schools adjacent to roads with moderate traffic activity were higher than those with low and very low traffic activity (as shown in Figures 1 and 2). This indicates that the outdoor sources of air pollution (particularly the traffic emission) have direct and considerable effects on the indoor level of air quality. On the contrary, the slightly higher mean level of CO _2_ in schools adjacent to the very low traffic street compared to those at the low traffic street, suggests that there is another source of CO _2_ inside the school itself, which will be discussed later.

To confirm the above results, the average concentrations of outdoor air pollutants were compared with those inside schools as (shown in Figures 3 and 4). Except for CO _2_, the average concentrations of all pollutants outside schools were slightly higher or nearly the same as the indoor levels, with no statistical differences for all pollutants (p > 0.05). This suggests that outdoor traffic activities are the main source of these pollutants inside schools and any indoor environments, such as homes. On the contrary, the higher mean level of CO _2_ inside the selected schools than its outdoor level suggests the predominance of the indoor source of this gas.

The findings of this study are comparable with similar studies inside and outside KSA. For example, a study conducted by Elsharkawy in several boys’ schools in Dammam and Khobar cities of the Eastern Province of KSA revealed that the highest levels of the same air pollutants inside classrooms that were located directly on the moderate traffic streets compared with low or very low traffic activity ones. It was also concluded that the average concentrations of studied air pollutants outside schools were slightly higher than those indoor, except CO _2_. ^26^ In Barcelona (Spain), impact of the outdoor traffic-related air pollutants was studied in 39 schools in an urban area with less traffic count and a heavy traffic area. The result of this study indicated that the concentration of air pollutants measured was higher in schools with heavy traffic activity, due to the higher influence of emission sources in the outdoor environment. ^27^ A recent study in Beijing (China) revealed that the air quality inside the school classrooms was greatly affected by the outdoor levels of air pollutants and the level of both PM _2.5_ and PM _10_ at most schools had adverse respiratory effects on children. ^28^ Another study was conducted in Coimbra, Portugal, indicated that the levels of CO _2_ in the indoor air in many schools were higher than those of outdoor air and most of these levels were above the recommended air quality guideline, particularly in the fall and winter seasons. ^29^ Many other studies conducted worldwide have revealed that in the absence of indoor sources of air pollutants, their concentrations indoors will be close to or lower than those outdoor because air pollution in the outdoor atmosphere is likely to be present indoors. ^30^

### Effect of the school building type on the IAQ levels

The independent t-test values indicated a statistically significant difference for CO, CO _2_, and NO _2_ levels (p < 0.05) between governmental constructed and rental school buildings, while there is no significance for the other pollutants. This could be attributed to the significant difference in the design of the two building types. School sizes, number of classrooms, and number of occupants in government school buildings are usually higher than the rental type. In addition, most governmental constructed buildings are located at or near traffic roads contrary to rental buildings that are usually located inside residential areas. Therefore, levels of air pollution inside the governmental constructed buildings were logically higher than rental buildings.

Reportedly, the above conclusion is the same in boys’ schools in KSA. ^26^ A similar study was conducted in 16 urban schools in the mid-Atlantic region in the US to determine the effect of the schools’ building type in the IAQ level. This study revealed that high concentrations of NO _2_ and CO _2_ were observed in temporary buildings schools compared to the permanent buildings’ schools. ^31^

### Effect of the classroom location on the IAQ levels

The location of the classroom inside any school may have an important role in the IAQ level inside it. This includes the floor number in which the classroom was located and the outside area which surrounds the classroom. Some of the studied classrooms for this study were located at the ground level (ground floor), while the others were located on the upper floors. The mean concentrations of pollutants inside classrooms that are located were higher than those of the upper floors without any significant differences for all pollutants as shown by the independent t-test analysis. Due to the dispersion process, the concentration of air pollutants decreases with increasing the horizontal and vertical distances, and for this reason, levels of pollutants on the upper floor were the lowest. In addition, the ground floor is closer to the traffic activity outside the school than the upper floor, which confirms the previous conclusion of the direct effect of the outside traffic activity on the IAQ inside schools and their classrooms. The indoor sources play an important role in the distribution of air pollutants in the school rooms. For instance, dust in the ground can be moved to the ambient air by the movement and playing of students in the playground, and hence, rooms on the ground floor can receive an amount of dust higher than the upper floors. Levels of the airborne particles strongly depend on particle size. The larger the particles are in terms of diameter, the heavier they are, and the more easily they can be deposited on the lower floor. ^32^

Similar results were obtained from many studies across the world. For instance, a study conducted in Upper Silesia, Poland, in 2015 revealed that the highest average concentrations of PM were obtained in all buildings and classrooms during occupancy periods located on the ground floor. The mean concentrations of PM _10_ and PM _2.5_ in classrooms of the ground floor were 166.12 and 125.69 μg/m ^3^, respectively, while their levels on the first floor were 81.49 and 67.65 μg/m ^3^, respectively. ^32^ In Dammam and Khobar cities in KSA, the mean concentrations of pollutants inside classrooms that are located at the first floor were higher than those of the upper floors in most schools. ^26^

### Effect of the ventilation quality inside classrooms on the IAQ levels

Generally, the use of CO _2_ as a marker for indoor air quality is widely used, and its level inside a building is usually used as indicator for the controlled ventilation. ^33^^,^^34^ To study the efficiency of ventilation rate inside classrooms, levels of CO _2_ were measured and linked with several factors during this study: the area of windows and doors related to the area of the classrooms and the number of students.

It was found that there was not any relation between the area of the classroom and concentration of CO _2_.This can be related to many factors such as the number of students inside the classrooms, ventilation rate, indoor exchange rate, and room design (e.g., floor area and room volume). ^35^ Similarly, there was no correlation between the total area of all windows and doors inside each classroom and level of CO _2_ (ppm). Natural ventilation is the intentional flow of outdoor air through an enclosure under the influence of wind and thermal pressures through controllable openings. Natural ventilations driven by pressure differences across the openings caused by ambient pressure and temperature differences between different openings within a unit. ^36^ On the other hand, the mechanical ventilation systems circulate fresh air using ducts and fans, rather than relying on airflow through small holes or cracks in a home’s walls, roof, or windows. In many cases, mechanical ventilation is very important to provide fresh air and prevent or reduce levels of moisture, odors, and other pollutants that can build up inside a home. ^37^ Unlike other Arab countries, such as Egypt, most of the buildings inside the KSA are completely dependent on the mechanical ventilation system or air conditioning system, despite the presence of windows and other openings inside these buildings. The main cause for this phenomenon is the climatic characteristic and the presence of high levels of dust in the environment of most KSA cities. For this reason, in KSA, most of the windows in schools and classrooms are completely closed, and they are used only for transmitting the sunlight, but not for natural ventilation. It is clear from the results of this study that there was no correlation between the number or volume of windows and the level of CO _2_ gas inside the selected classrooms, which indicates that levels of air pollutants inside the classroom depend mainly on the efficiency of mechanical and not natural ventilation systems.

Because the human body is considered a source of CO _2_ in the atmosphere as the result of the expiration process, a correlation between numbers of students and levels of this gas was studied inside each classroom. Unexpectedly, no correlation was found between number of students and level of CO _2_ in the classrooms, as shown in Figure 9. This may be related to the ventilation rate and air conditioning system, which was applied inside each classroom. These factors are considered one of the most important control techniques that are used for improving the IAQ inside any closed room. The low levels of CO _2_ with the high number of students in some classrooms can be explained by the good ventilation system and rate inside these classrooms.

Many previous studies revealed that the high levels of CO _2_ in schools are correlated with high occupancy or inadequate ventilation. For example, a study conducted in the UK indicated that an elevated level of CO _2_ rose quickly to 3000– 4500 ppm when windows were left closed in the absence of other means of providing air, such as conditioning. ^38^ Another study conducted in Victoria, Australia, during the winter periods indicated that a high level of CO _2_ (2700 ppm) was recorded in typical occupied and non-ventilated rooms. ^39^

### Effect of the temperature and RH on IAQ levels

Temperature is one of the basic IAQ measurements that has a direct impact on perceived comfort and in turn, concentration, and productivity. According to the American Society of Heating, Refrigerating and Air Conditioning Engineers Standard (ASHRAE), the recommended temperature ranges perceived as “comfortable” are 73°F –79°F (22.8°C–26.1°C) in the summer and 68°F –74.5°F (20.0°C–23.6°C) in the winter. ^40^ If the air temperature is too hot or too cold, this will make the occupants uncomfortable, and it would probably make them use only half of their mind and effort in concentrating on their job while the other half is concentrating on the uncomfortable air quality. ^41^ During this study, the temperature degrees inside classrooms of all schools ranged between 20.1°C–25.5°C, which is consistent with the recommended values. Results of our study are comparable to a study conducted in south-western school districts in the US to monitor the temperature inside the classrooms with closed windows and doors and ventilation rates operated by mechanical ventilation. It was concluded that there was no statistically significant correlation between indoor temperature and CO _2_, while there was only a weak positive correlation between indoor and outdoor average temperature (Spearman correlation.243, p < 0.05) indicating that indoor temperature is relatively independent of the outdoor conditions. ^42^

The IAQ and thermal comfort also depend greatly on the humidity level, which enhances the microbial growth, increases heat index, and the level of the fine airborne particles. For example, the human body feels warmer in humid conditions, and high temperatures affect and worsen people’s health. ASHRAE recommends the acceptable range of RH is 30%– 60% for all seasons. ^43^ Relative humidity below 30% is unacceptable because it can cause irritation and could contribute to symptoms such as eye and nose irritation, headache, cough, dry facial skin, and difficulties in breathing. RH above 60% may support the growth of pathogenic or allergenic microorganisms and more contribute to the impact of heat index. ^44^ In the current study, the range of RH percentage was (from 29 % to 90.1%). The Pearson correlation coefficient test indicated that except VOCs, there was a weak to moderate positive correlation between RH and all pollutants with a strong statistical significance difference (p = 0). As for VOCs, there was a very weak negative correlation without statistical significance for all pollutants (p ˃ 0.05), as shown in Table 4. In Finland, low RH (21%–23%) was recorded inside all classrooms of six public schools. ^45^

### Effect of furnishing products on the IAQ levels

Many air pollutants are emitted in the indoor atmosphere from the furnishings, where several materials are used such as wood, textile, plastics and metals. For example, VOCs are emitted from paints, solvents, and color-painted wood. ^46^ Most of these products are bonded with urea-formaldehyde adhesive, which leads to off-gassing of formaldehyde due to chemical reactions during their service lives. ^47^ Inside schools, there is much furniture of different types including classroom desks, chairs, cabinets, tables, and others. One of the major sources that contributed to the build-up of VOCs indoor environment is indoor emissions from furniture and building materials. ^48^ To study the effect of furnishing products in schools, the level of VOCs was correlated with the amount of furniture at each measuring site inside the school (classrooms, teachers’ rooms, and administrative offices). Generally, levels of measured VOCs were small (0.1–1.05 mg/m ^3^) without any correlation with the number of furniture, which means that the furnishing products have no measurable participation in these levels. The Pearson correlation test indicated that there was a very weak negative correlation between the number of furniture and levels of VOCs. A similar study was conducted in 144 classrooms at 37 recently constructed or renovated schools in the US. The median concentration among the most observed VOCs inside classrooms (benzene and toluene) were 0.3 and 0.7 mg/m ^3^, respectively, and most mean concentrations of VOCs were below 5 mg/m ^3^ ^49^ which is inconsistent with this present study.

### Air quality guidelines (AQG)

It is very important to compare the results of the IAQ studies with the Air Quality Guidelines (AQGs) that represent acceptable limits for the air pollutants recommended by the governmental authorities of each country. ^50^ Unfortunately, there are no IAQ guidelines in the Saudi Environmental Law. Results of this study were compared with the indoor AQGs suggested by different international scientific agencies ( Table 6), such as the U.S. Environmental Protection Agency (USEPA), the WHO, the National Institute for Occupational Safety and Health (NIOSH), the Occupational Health and Safety Administration (OSHA), the American Conference of Governmental Industrial Hygienists (ACGIH) and ASHRAE (NAAQS/EPA, 2006; OSHA; NIOSH, 1992; ACGIH, 2001; ASHRAE, 2010). ^44^^,^^51^^–^^55^ As shown in Table 7, comparing the results of this study with their AQGs indicated that the average levels of air pollutants inside the studied schools were much lower than their AQGs, while some levels of CO _2_ and NO _2_ were exceeding their AQGs at some schools.

**Table 6.** International air quality guidelines (AQG) for common indoor pollutants.

Pollutants	NAAQS/EPA	OSHA	WHO/Europe	NIOSH	ACGIH	ASHRAE
PM _10_ (μg/m ^3^)	150 (24 hr) 50 (1 yr)		50 (24 hr)		1000 (8 hr)	
PM _2.5_ (μg/m ^3^)	35 (24 hr) 15 (1 yr)	5000 (8 hr)				
CO (ppm)	9 (8 hr) 35 (1 hr)	50 (8 hr)	10 (8 hr) 25 (1 hr) 50 (30 min) 90 (15 min)	35 (8 hr)	25 (8 hr)	
CO _2_ (ppm)		5000 (8 hr)		5000 (8 hr) 30000 (15 min)	5000 (8 hr) 30000 (15 min)	1000 (8 hr)
NO _2_ (ppm)	0.05 (1 yr)		0.1 (1 hr) 0.004 (1 yr)	1.0 (15 min)	3 (8 hr) 5 (15 min)	
VOCs (μg/m ^3^)	200-600					

**Table 7.** Overall mean levels of air pollutants inside the selected schools.

School No.	PM _10_ (μg/m ^3^)	PM _2.5_ (μg/m ^3^)	CO _2_ ppm	CO ppm	NO _2_ ppm	VOCs ppm
1	29.3	15.8	1107.4 *	3.85	1.32 *	0.3
2	17	11.1	723.5	3.73	0.58 *	0.23
3	17.3	8.8	531.8	3.7	0.28 *	0.16
4	21.9	14.4	1488 *	2.86	0.73 *	0.29
5	16.3	11.6	691.3	2.95	0.56 *	0.10
6	15.25	8.5	702.5	1.925	0	0.34
7	15	12.8	580.3	2.73	0	0
8	12	9	614.8	2.63	0	0
9	14.3	12	523	2.2	0	0
10	18	9.5	672.8	2.9	0	0
11	16.8	12.3	601.8	2.23	0	0
12	28.8	14	951.8	2.05	0	0.25
13	23.8	13.8	685	1.58	0.91 *	0
14	24.8	16.3	1019.8 *	1.86	0.915 *	0.28
15	20.8	13.5	669.5	1.2	0.23 *	0
16	14.8	7.5	653.3	2.6	0.25 *	0
17	15.5	11.5	561	2.13	0.38 *	0

* Levels of air pollutants that are exceeded the recommended AQG.

Measurements of CO _2_ concentrations inside 60 schools in Croatia revealed that all recorded levels were higher than the recommended air quality guidelines (1938 mg/m ^3^), where the levels ranged between 2771 and 7763 mg/m ^3^ due to the poor ventilation in the classrooms, particularly in the hot months. ^56^ In Wellington, the capital of New Zealand, a study was done to analyse the concentration and sources of air pollution at an urban primary school (5–11 years), where the indoor PM _10_ mean concentrations during the school day (30.1 μg/m ^3^) were significantly (p < 0.001) higher than the mean outdoor concentrations (8.9 μg/m ^3^), and the primary driver of indoor PM _2.5_ was from the infiltration of outdoor pollutants from the motor vehicle emissions. ^57^

In Kuwait, a study was conducted to assess IAQ during a complete school calendar year and covered all climatic seasons. IAQ parameters were examined to assess pollutant levels in Kuwait schools in multiple settings (classrooms, painting rooms, computer labs, science rooms, teachers’ rooms, and roofs). The study revealed that high concentrations of VOCs and dust were present in most schools. ^58^

Another study was done in five primary schools in the Maltese Islands. The mean indoor PM _2.5_ level of 17.78 μg/m ^3^ and CO (9.11 ppm) exceeded the thresholds set by the WHO. ^59^ The Park study showed the effects of outdoor temperature on the exam day on student performance, using 4.6 million high school exit tests in New York. The author finds that students taking an exam on a day with temperatures higher than 32°C scores up to 14% lower. ^60^

Another study was done in the primary schools of six French cities to study the poor air quality in classrooms, where the mean concentration of NO _2_ has exceeded the WHO guidelines. ^61^ In 18 schools located in urban, industrial, and rural areas in Central–Southern Spain, the NO _2_ concentrations were higher in an urban area with mean ranging between (17.6 and 113.0 mg/m ^3^) and followed by schools located in industrial or near highway means between (12.9 and 32.1 mg/m ^3^) than those in rural with mean ranging between (6.30 and 13.9 mg/m ^3^). ^62^

### Strengths and limitations of the study

The Arab governmental schools are usually located in areas of heavy air pollution sources (e.g., congested traffic and industry). Hence, the air pollution level inside these schools is expected to be high. Generally, there is lack of research in this field. The major importance of this study is the formation of a database for air quality, inside Saudi governmental schools as a representative to other Arab countries. In addition, this study will help in raising awareness of students and all school personnel concerning the environmental health, especially maintaining good air quality.

The main limitation of this study was the number of schools. It was planned to monitor a higher number, but unfortunately this was limited due to the coronavirus disease 2019 (COVID-19) pandemic where the learning process has completely shifted to distance education (online). The nature of this study requires the daily visiting of schools to conduct the measurements.

## Conclusion

Levels of the six studied air pollutants inside schools adjacent to roads with moderate traffic activity were higher than those with low and very low traffic activity, indicating that outdoor sources of air pollution (particularly traffic emissions) have direct and considerable effects on the IAQ level. In addition, except for CO _2_, the mean levels of other pollutants outside schools were slightly higher or nearly the same as the indoor levels, confirming that the outdoor traffic activity is the main source of these pollutants inside schools or any indoor environments. On the contrary, the mean level of CO _2_ inside the studied schools was higher than its outdoor level, suggesting the predominance of the indoor source of this gas.

Due to the dispersion process, the concentration of air pollutants decreases with increasing the horizontal and vertical distances, and thus levels of pollutants on the upper floors were lower than those on the ground floors. In addition, the ground floor is closer to the traffic activity outside the school than the upper floor, which supports the previous conclusion of the direct effect of outside traffic activity on the IAQ level inside schools and their classrooms. Levels of air pollutants inside all studied schools were much lower than their AQGs, while some CO _2_ and NO _2_ levels exceeded their AQGs in some schools.

## Data availability

### Underlying data

Figshare: Full data.xlsx. https://doi.org/10.6084/m9.figshare.19403078.v1. ^63^

The file contains the following underlying data:

• Full details of the selected schools for this study
• Monitoring sites inside the selected schools
• Levels of air pollutants at each selected site

Data are available under the terms of the Creative Commons Attribution 4.0 International license (CC-BY 4.0).
