# Supplementary material for: Indoor air quality at the Arab governmental girls’ schools
Source: F1000Res. 2024 May 30;11:1125. Originally published 2022 Sep 29. [Version 2] doi: 10.12688/f1000research.110775.2 (PMC11776455; doi:10.12688/f1000research.110775.2)
Supplement: Peer review history

# Reviewer report: Reviewer response for version 2

**DOI:** 10.5256/f1000research.139283.r358050

**Published:** 2025-01-28

**Authors:** Katarzyna Gładyszewska-Fiedoruk

**Affiliations:** 1 Department of Hydraulics and Sanitary Engineering, Faculty of Civil and Environmental Engineering, Institute of Environmental Engineering, Warsaw University of Life Sciences (SGGW), Warsaw, Poland

Very extensive study. It begs to be modeled, correlated, etc. Generally processed mathematically. I highly recommend creating another article with a mathematical analysis of pollutions. The articles presenting the experiments must contain a thorough formal analysis of experimental errors, including errors in all measured variables and parameters presented, description of the instruments used, how they are used, calibration, etc. reality and overall error calculation in all applied maintenance and other rules.

Is the work clearly and accurately presented and does it cite the current literature?

Yes

If applicable, is the statistical analysis and its interpretation appropriate?

Not applicable

Are all the source data underlying the results available to ensure full reproducibility?

Yes

Is the study design appropriate and is the work technically sound?

Yes

Are the conclusions drawn adequately supported by the results?

Yes

Are sufficient details of methods and analysis provided to allow replication by others?

Yes

Reviewer Expertise:

IAQ

I confirm that I have read this submission and believe that I have an appropriate level of expertise to confirm that it is of an acceptable scientific standard.

# Reviewer report: Reviewer response for version 2

**DOI:** 10.5256/f1000research.139283.r284918

**Published:** 2024-08-14

**Authors:** Eltigani Omer

**Affiliations:** 1 Northern Boarder University, Arar, Saudi Arabia

All required correction done

 Thank you

Is the work clearly and accurately presented and does it cite the current literature?

Yes

If applicable, is the statistical analysis and its interpretation appropriate?

Yes

Are all the source data underlying the results available to ensure full reproducibility?

Yes

Is the study design appropriate and is the work technically sound?

Yes

Are the conclusions drawn adequately supported by the results?

Yes

Are sufficient details of methods and analysis provided to allow replication by others?

Yes

Reviewer Expertise:

Environmental Epidemiology, Public Health

I confirm that I have read this submission and believe that I have an appropriate level of expertise to confirm that it is of an acceptable scientific standard.

# Reviewer report: Reviewer response for version 2

**DOI:** 10.5256/f1000research.139283.r263752

**Published:** 2024-08-07

**Authors:** Adel Zakaria

**Affiliations:** 1 High Institute of Public Health - Alexandria University, Alexandria, Egypt

Dear Author,

 Thank you a lot for your kind words. I spent some time and effort to review your paper aiming to critically and subjectively find out any observations. You have already corrected some of them and replied for the others. However, I was not convinced by some of your replies. Hereon I will comment on those replies. (You can refer to my original report for Version 1 here.)

 I missed in my previous report the original research data. However I found it on the Excel sheet. I screened through it and found it to be okay.

 I read the manuscript for the third time, in general, as I mentioned before in my previous report, the work is a good one, however it needs some refinements to be much better.

 I will comment on the replies which I didn’t accept them.

**Minor Mistakes:**

 Mention of RH on the comments of Table 6.

 Reply: The authors said that they mentioned it under the table

 Comment: You might mention it in table title, however not on the comments on the table

 Reply: Concerning ventilation on the second paragraph, you mentioned the importance of ventilation and a comparison between natural and mechanical ventilation in details.

 Comment: Natural ventilation is not applied in your research at all, so there isn't any sound reason to mention it in details and make a comparison between it and mechanical ventilation.

 Reply: Concerning interpretation of the unrelated CO _2_concentration with number of students in the classroom.

 Comment: The authors may have misinterpreted my reservation. It needs to be clearly interpreted.

 The interpretation in the manuscript “third paragraph under Ventilation in discussion” is based on theoretical basis and assumption and not by practical measurements and calculations.

**Serious Mistakes:**

 Reply: Concerning the title, the authors have emphasized to keep it the same.

 Comment: However, your reply is not sound enough, since adding “in KSA” at the end of the title will be more attractive for the research to be applied in other Arab Countries, also there are thousands of research papers where the place or country is added, which I believe it is essential, in contrary to what you said.

 Reply: How did the reviewer conclude that the research was extracted from a thesis?

 Comment: The author said from” The Thesis” and not from a thesis which emphasizes that the present research as well as the safety survey research were extracted from “The Thesis” since the two researches were done at the same schools on the same time. Also the safety survey research was published after IAQ research by about a year, so how it comes that the safety survey content was mentioned in IAQ research.

 It seems that may be Ms. Sana is the thesis candidate, since her name is on the two researches.

 Besides, the two researches were massive surveys which needs a lot of time and efforts so that it seems to be difficult for the faculties to do it alone, however, the thesis candidate would accommodate himself/herself to implement the research and recruit some assistants.

**Results**

 Reply: Concerning putting the IAQ guidelines and occupational exposure limits in the same table (Table 6).

 Comment: Your reply is theoretically right, however, in the entire manuscript there was no signal at all to occupational exposure of school staff, and the whole study was dedicated to study IAQ in the chosen schools. If you have noticed the remarkable difference between IAQ guidelines and occupational exposure limits, and that the results of the study were very near to IAQ guide lines or even much less.

 So that, the occupational exposure limits should be omitted from Table 6.

**Discussion:**

 Reply: The authors' reply concerning “Effect of school building type on the IAQ levels” is not related to my reservation in my previous report. You didn’t reply to my concern about discussing the impact of two parameters together on the IAQ, under that title you should discuss only the impact of school building type on IAQ.

 As I discussed before, the authors haven't written the same explanation in their reply about the school building type in the original manuscript or add it to your modified version.

 A better discussion of that parameter declaring its solely impact on IAQ is highly recommended.

Is the work clearly and accurately presented and does it cite the current literature?

Yes

If applicable, is the statistical analysis and its interpretation appropriate?

I cannot comment. A qualified statistician is required.

Are all the source data underlying the results available to ensure full reproducibility?

No

Is the study design appropriate and is the work technically sound?

Yes

Are the conclusions drawn adequately supported by the results?

Yes

Are sufficient details of methods and analysis provided to allow replication by others?

Yes

Reviewer Expertise:

Occupational Hygiene, Safety, Air Pollution Control

I confirm that I have read this submission and believe that I have an appropriate level of expertise to confirm that it is of an acceptable scientific standard, however I have significant reservations, as outlined above.

# Reviewer report: Reviewer response for version 1

**DOI:** 10.5256/f1000research.122419.r261827

**Published:** 2024-05-21

**Authors:** Adel Zakaria

**Affiliations:** 1 High Institute of Public Health - Alexandria University, Alexandria, Egypt

Dear Sir

 Greetings to you from Alexandria,

 Thank you for allowing me to review this article.

 I apologize, I won't summarize the article.

 I read it critically, and found some minor mistakes and some serious mistakes.

 I will start with the minor ones then the serious ones.

 I will follow a systematic approach in my review.

**General overview:**

 The article is a good one, written in a sound language, with minimal spelling or grammar mistakes.

 Each section of the research paper is covering its subject properly, the methodology and instruments used is quite proper for sampling and  monitoring of air pollutants.

 the results is presented in a good manner in tables and figures, and discussion is detailed and succeeded in interpreting the results and supported it with recent literature.

 The references is more than quite enough and more than half of them are considered recent.

 However, it seems that this paper is extracted from a thesis, which will be clarified later.

**Minor Mistakes:**

 Under the title" Monitoring of IAQ" in the sixth line (PM10 and PM2.5 respectively) the order should be reversed.

 In paragraph after Fig. 4, the sixth line statement starting by" The independent t-test....." is repeated in the eighth line.

 The comments on Table 5 didn't contain any mention to RH, despite it is mentioned in the table.

 Under the title "effect of Ventilation" second paragraph is too long and discussed in details definition of natural and mechanical ventilation and the difference between them. Its better to be summarized.

 The third paragraph is not convincing to interpret the unrelated CO _2_ concentration with number of students in the classroom. It needs to be interpreted.

 The paragraph after Table 7 the gas concentration is mentioned in mg/m ^3^ its better to convert it to ppm like the present paper.

 Under the title " Strengths and limitations of the study" in the fourth line "as a representative of to other Arab countries" it is better to  be Gulf countries.

**Serious Mistakes**:

**The Title**, I believe that Its better to add ": Al-Qatif-KSA" to the title, at the end of it.

**Abstract**, ****the last statement in the Methods," a predesigned questionnaire was used to evaluate the safety preparedness in the same selected schools"

 There was no mention at all to the questionnaire or its results in the entire paper, emphasizing that the present paper extracted from a thesis., also at the end of introduction the last statement there is a mention of safety. However, there is no mention in Methodology to any safety preparedness plan.

**Results**: In table 6, it is not valid to put limits of NIOSH and ACGIH in this table of AQG since their limits is designated to working environment.

**Discussion:**Under the title" Effect of the school building type on the IAQ levels" The reason that the levels of increased levels of pollutants in governmental schools are greater  than those in the rented ones is not clear. The authors mentioned theoretical reasons with no relation to that difference. Also they combined another parameter, which is the location nearby high or low traffic  areas which confuses the readers. Also the American reference at the third paragraph didn't offer an explanation since it was comparing between temporary and permanent schools.

 Under the title"Effect of temperature& relative humidity on IAQ levels", the authors discussed the effect of temperature and RH separately, however its better to consider the "effective temperature"

 Yet, these are the most important comments on the present paper, on correction of these comments, I believe that the paper will be better.

 Dr. Adel Zakaria

 Prof. of Occupational Hygiene and Air Pollution 

 Alexandria University

 Alexandria- Egypt

Is the work clearly and accurately presented and does it cite the current literature?

Yes

If applicable, is the statistical analysis and its interpretation appropriate?

I cannot comment. A qualified statistician is required.

Are all the source data underlying the results available to ensure full reproducibility?

No

Is the study design appropriate and is the work technically sound?

Yes

Are the conclusions drawn adequately supported by the results?

Yes

Are sufficient details of methods and analysis provided to allow replication by others?

Yes

Reviewer Expertise:

Occupational Hygiene, Safety, Air Pollution Control

I confirm that I have read this submission and believe that I have an appropriate level of expertise to confirm that it is of an acceptable scientific standard, however I have significant reservations, as outlined above.

# Response

**Published:** 2024-05-25

**Authors:** Mahmoud Elsharkawy

**Affiliations:** Imam Abdulrahman Bin Faisal University, Saudi Arabia

**Dear Reviewer**

 First, I would like to greatly thank you for your valuable comments that improved the manuscript and gave it more strength and reliability. We considered all your comments in the revised manuscript as follows: 

**Minor Mistakes:**

1. Under the title" Monitoring of IAQ" in the sixth line (PM10 and PM2.5 respectively) the order should be reversed.

Response

 We reversed the order.

  

1. In paragraph after Fig. 4, the sixth line statement starting by" The independent t-test....." is repeated in the eighth line.

Response

 We deleted the repeated sentence.

  

1. The comments on Table 5 didn't contain any mention to RH, despite it is mentioned in the table.

Response

 We already mentioned it under the table.

  

1. Under the title "effect of Ventilation" second paragraph is too long and discussed in details the definition of natural and mechanical ventilation and the difference between them. Its better to be summarized.

Response

 Ventilation is a very important factor in the indoor environment. Not all readers have the same experience with the detail of this factor. We found that it is important to detail.

  

1. The third paragraph is not convincing to interpret the unrelated CO2 concentration with the number of students in the classroom. It needs to be interpreted.

Response

 We already mentioned that the use of CO2 as a marker for indoor air quality is widely used, and its level inside a building is usually used as an indicator for controlled ventilation.

  

1. The paragraph after Table 7 the gas concentration is mentioned in mg/m3 its better to convert it to ppm like the present paper.

Response

 It will not make any difference because the two units are used for air pollutants.

  

1. Under the title " Strengths and limitations of the study" in the fourth line "as a representative of to other Arab countries" it is better to be Gulf countries.

Response

 We changed it.

**Serious Mistakes:**

1. The Title, I believe that Its better to add ": Al-Qatif-KSA" to the title, at the end of it.

Response

 We used the Eastern Province of Saudi Arabia as a representative to most Arab cities. So, to make our paper valuable and can be widely used in Arab countries, we used this title like thousands of papers worldwide.

  

1. Abstract, the last statement in the Methods," a predesigned questionnaire was used to evaluate the safety preparedness in the same selected schools".

Response

 We deleted this statement from the abstract.

  

1. There was no mention at all to the questionnaire or its results in the entire paper, emphasizing that the present paper extracted from a thesis., also at the end of introduction the last statement there is a mention of safety. However, there is no mention in Methodology to any safety preparedness plan.

Response

- How did the reviewer conclude that the research was extracted from the thesis? We have already conducted a safety survey, but its results have been published in independent research. No doubt that providing a safe and clean environment inside schools will consider an important “ **safety** procedure” for the students and school staff.
- However, we deleted the “safety preparedness issues” from the paper.

 

1. Results: In table 6, it is not valid to put limits of NIOSH and ACGIH in this table of AQG since their limits is designated to working environment.

Response

 The school environment is considered as an indoor environment for (for students as examples) and a workplace for all staff (managers, teachers, administrative workers……).

  

1. Discussion: Under the title" Effect of the school building type on the IAQ levels" The reason that the levels of increased levels of pollutants in governmental schools are greater than those in the rented ones is not clear. The authors mentioned theoretical reasons with no relation to that difference. Also they combined another parameter, which is the location nearby high or low traffic areas which confuses the readers. Also the American reference at the third paragraph didn't offer an explanation since it was comparing between temporary and permanent schools.

Response

- We described the reality of the situation of schools, as public (or governmental) schools are usually characterized by their large capacity and location that is qualified to receive a large number of cars, their multiple floors, and high number of students and related activities (labs, large playground…….etc.). This is completely different from rental schools that belong to a person or group of persons who rent a spacious residence and turn it into a school.
- As for the American study, it referred to the same difference, but with different names.

 

1. Under the title "Effect of temperature& relative humidity on IAQ levels", the authors discussed the effect of temperature and RH separately, however its better to consider the "effective temperature".

Response

 We changed the temperature to “effective temperature”.

# Reviewer report: Reviewer response for version 1

**DOI:** 10.5256/f1000research.122419.r266512

**Published:** 2024-04-25

**Authors:** Eltigani Omer

**Affiliations:** 1 Northern Boarder University, Arar, Saudi Arabia

- Clearly and accurately presented and it cites the current literature and the references were appropriate, so the authors were able to fully address the topic.
- Ethically it was approved by the Institutional Review Board (IRB) of Imam Abdurrahman bin Faisal University No. IRB-PGS-2019-03-357 at 4/12/2019.
- More detail is needed about measuring the levels of air pollutants and method followed to select the study units (schools).
- Please provide evidence that the traffic activity in Al-Qatif is not congested compared to other cities of the Eastern Province of KSA as you reported?
- The average number of cars that moving per hour varies at different times; please explore how you avoid this potential error?
- Explore the method followed to select measuring sites inside the school.
- The results were well presented and appropriate statistical measures were used to determine the significance of association between study variables.
- The various indoor sources that influenced the contribution and concentration of pollutants need more exploring.

Some references listed below are very old and need to be updated:  

- Borse NN, Gilchrist J, Dellinger AM, *et al.*: CDC childhood injury report: Patterns of unintentional injuries among 0- to 19-year-olds in the United States, 2000–2006. *J Fam Community Med.* 2008; **32**(2): 189.
- Baldauf R, Watkins N, Heist D, *et al.*: Near-road air quality monitoring: Factors affecting network design and interpretation of data. *Air Qual Atmos Health.* 2009; **2**: 1–9. Publisher Full Text
- Leong ST, Muttamara S, Laortanakul P: Preliminary study of relationship between outdoor and indoor air pollutant concentrations at Bangkok’s major streets. *Sci Technol Asia.* 2003; **8**(3): 29–39. Accessed January 27, 2021. Reference Source
- NAAQS/EPA-U.S. Environmental Protection Agency: *Code of Federal Regulations, Title 40 CFR, Part50 National Ambient Air Quality Standards.* 2006.
- National Institute for Occupational Safety and Health: *NIOSH recommendations for occupational safety and health – Compendium of policy documents and statements.* Diane Publishing;1992.
- ACGIH (American Conference of Governmental Industrial Hygienists): Threshold limit values for chemical substances and physical agents and biological exposure indices.2001.
- Elsharkawy M: Full data.xlsx. figshare. [Dataset].2022. Publisher Full Text

Is the work clearly and accurately presented and does it cite the current literature?

Yes

If applicable, is the statistical analysis and its interpretation appropriate?

Yes

Are all the source data underlying the results available to ensure full reproducibility?

Yes

Is the study design appropriate and is the work technically sound?

Yes

Are the conclusions drawn adequately supported by the results?

Yes

Are sufficient details of methods and analysis provided to allow replication by others?

Yes

Reviewer Expertise:

Public Health, Environmental Epidemiology

I confirm that I have read this submission and believe that I have an appropriate level of expertise to confirm that it is of an acceptable scientific standard.
